# From Transverse Myelitis to Optic Atrophy: An Unfavorable Clinical Course of AQP4-Positive Neuromyelitis Optica With Onset During Pregnancy and Multiple Relapses

**DOI:** 10.7759/cureus.90846

**Published:** 2025-08-23

**Authors:** Lizeth Valeria Zuluaga Gómez, Christian D Messu Llano

**Affiliations:** 1 Medicina, Unidad Central del Valle, Tuluá, COL; 2 Internal Medicine, Hospital Universitario del Valle, Cali, COL; 3 Internal Medicine, Universidad del Valle, Cali, COL

**Keywords:** aqp4 antibodies, azathioprine, immunosuppressive therapy, longitudinally extensive transverse myelitis, neuromyelitis optica, optic neuritis, plasmapheresis, postpartum relapse, pregnancy, rituximab

## Abstract

Neuromyelitis optica spectrum disorder (NMOSD) is an autoimmune inflammatory disorder of the central nervous system that primarily affects young women and is associated with antibodies against aquaporin-4 (AQP4). Disease onset during pregnancy presents both diagnostic and therapeutic challenges, and there is an increased risk of relapse in the postpartum period, which can lead to a higher risk of long-term sequelae. We present the case of a 22-year-old pregnant woman who developed longitudinally extensive transverse myelitis during the second trimester as the first manifestation of NMOSD and experienced multiple neurological relapses during follow-up, resulting in optic nerve atrophy as a permanent sequela. This case highlights the complex interaction between autoimmune disease, pregnancy, immunosuppressive therapies, and access barriers to treatment, emphasizing the importance of a multidisciplinary approach and sustained therapeutic adherence to prevent disability progression.

## Introduction

Neuromyelitis optica (NMO), a subtype of neuromyelitis optica spectrum disorder (NMOSD), is an autoimmune inflammatory disease characterized by severe attacks involving the optic nerves, causing neuritis, and the spinal cord, causing longitudinally extensive transverse myelitis (LETM), commonly associated with antibodies against aquaporin-4 (AQP4). This condition predominantly affects young women, and its onset during pregnancy presents unique clinical challenges, including an increased risk of obstetric complications such as spontaneous abortion, preeclampsia, and premature rupture of membranes [1].

Recent studies have shown that relapse rates double in the postpartum period compared to during pregnancy (annualized relapse rate of 1.0 postpartum vs. 0.2 pre-pregnancy, with 76.9% of relapses occurring within the first 12 postpartum months) [1,2]. Recent evidence supports the safety of azathioprine and rituximab during pregnancy, as well as corticosteroids to reduce disease activity and prevent perinatal attacks [2,3]. Successful use of newer therapies such as satralizumab during pregnancy and breastfeeding without neonatal adverse events has also been reported [4].

We present the case of a pregnant woman with NMO who presented with LETM at disease onset, experienced multiple postpartum relapses, and had interruptions in immunosuppressive therapy due to a combination of personal choice, administrative barriers, and therapeutic failure, ultimately resulting in optic nerve atrophy. This case highlights the complex interplay between pregnancy, immunosuppression, and treatment accessibility in clinical outcomes of patients with NMO.

## Case presentation

A 22-year-old pregnant woman at 14 weeks of gestation, without any relevant past medical history, presented to the emergency department with a one-week history of progressive, ascending weakness of the lower limbs, accompanied by electric shock-like pain originating in the cervical region and radiating to the thoracic and lumbar spine, and had experienced constipation and urinary retention for the past three days.

Neurological examination on admission showed an alert and oriented patient, with intact cranial nerves. Sensory testing showed bilateral hypoesthesia at the T4 level and anesthesia below T12. Muscle strength was 4/5 in the upper limbs, 2/5 in the left lower limb, and 1/5 in the right lower limb. Deep tendon reflexes were normal in the upper limbs, but there was bilateral patellar and ankle hyperreflexia, along with bilateral Babinski sign. A distended bladder was noted, and digital rectal examination revealed anal sphincter atony.

Initial laboratory tests, including biochemical markers and screening for chronic infections, were within normal limits (Table 1).

**Table 1:** Biochemical and chronic infection tests at admission. HIV, human immunodeficiency virus; HTLV, human T-lymphotropic virus; VDRL, Venereal Disease Research Laboratory

Test	Result	Reference range
Leukocytes	10.88 x 10^3^ cells/mm^3^	3.98-10.04 x 10^3^ cells/mm^3^
Neutrophils	8.69 x 10^3^ cells/mm^3^	1.56-6.13 x 10^3^ cells/mm^3^
Lymphocytes	1.62 x 10^3^ cells/mm^3^	1.18-3.74 x 10^3^ cells/mm^3^
Hemoglobin	15.1 g/dL	11.2-15.7 g/dL
Platelets	204 x 10^3^ cells/mm^3^	182-369 x 10^3^ cells/mm^3^
Creatinine	0.42 mg/dL	0.52-1.04 mg/dL
Sodium	134 mg/dL	134-146 mg/dL
Potassium	3.6 mg/dL	3.5-5.3 mg/dL
Calcium	8.82 mg/dL	8.5-10.2 mg/dL
Magnesium	1.9 mg/dL	1.7-2.2 mg/dL
Phosphorus	2.9 mg/dL	2.5-4.5 mg/dL
C reactive protein	<5 mg/dL	<5 mg/dL
Procalcitonin	0.138 ng/mL	<0.25 ng/mL
HIV antibodies	Negative	Negative
VDRL	Non-reactive	Non-reactive
Hepatitis B surface antigen	Negative	Negative
Hepatitis C total antibodies	Negative	Negative
HTLV I-II antibodies	Negative	Negative

**Figure 1:**
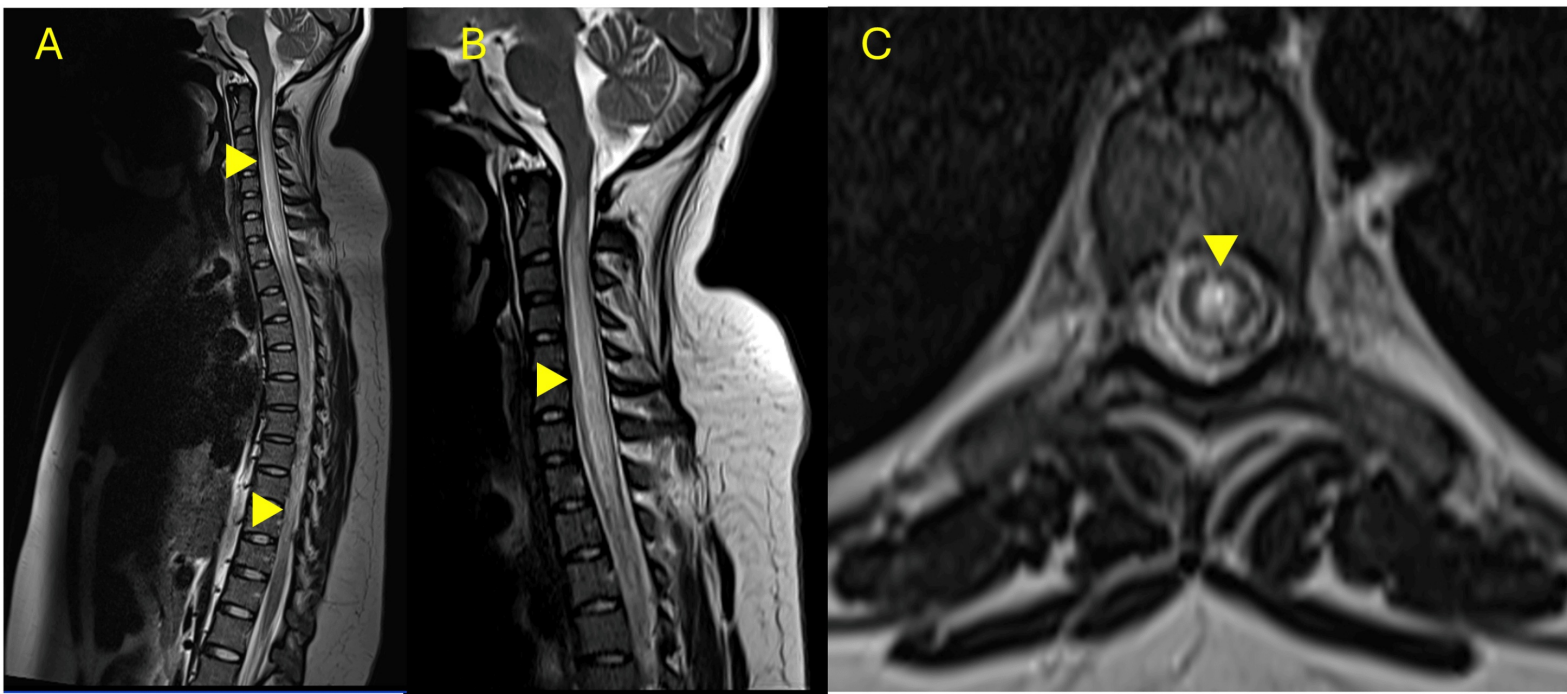
Spinal MRI findings at initial presentation showing LETM. A. Sagittal T2-weighted MRI of the cervicothoracic spine shows increased signal intensity and swelling of the spinal cord extending from C1–C2 to T11–T12 (yellow arrowheads), consistent with LETM. B. Sagittal T1-weighted MRI with gadolinium contrast demonstrates mild, homogeneous enhancement of the cervical lesion (yellow arrowhead), without evidence of mass effect or hemorrhage. C. Axial T2-weighted image at the thoracic level reveals central hyperintensity within the spinal cord (yellow arrowhead), indicating intramedullary involvement. LETM, longitudinally extensive transverse myelitis

A full spinal MRI revealed increased volume and T2 signal intensity of the spinal cord from C1-C2 to T11-T12, with mild, homogeneous contrast enhancement without diffusion restriction (Figure 1). These findings were consistent with LETM.

Lumbar puncture at admission revealed normal opening pressure. Cerebrospinal fluid (CSF) analysis showed mixed pleocytosis with a predominance of polymorphonuclear cells, elevated CSF protein levels, and mild hypoglycorrhachia. Based on these findings, empirical antibiotic therapy with cefepime and vancomycin was initiated. However, after multiple molecular and microbiological tests performed on a second CSF sample obtained 72 hours later, infectious etiologies were ruled out. Intravenous methylprednisolone and azathioprine (2 mg/kg/day) were then initiated due to high clinical suspicion of an autoimmune etiology. A weakly positive result for anti-AQP4 antibodies in the CSF confirmed the diagnosis of NMO (Table 2).

**Table 2:** Cerebrospinal fluid findings at initial presentation and follow-up AQP4, aquaporin-4 antibodies detected by indirect immunofluorescence; CSF, cerebrospinal fluid; PCR, polymerase chain reaction

	CSF #1	CSF #2	Reference range
Opening pressure	5 cm H₂O	10 cm H₂O	<20 cm H₂O
Appearance	Slightly turbid	Clear	Clear
Glucose	44 mg/dL	61 mg/dL	50–80 mg/dL
Proteins	210 mg/dL	69 mg/dL	<45 mg/dL
Red blood cells	27 cells/mm³	950 cells/mm³	<5 cells/mm³
Polymorphonuclear cells	345 cells/mm³	50 cells/mm³	<5 cells/mm³
Lymphocytes	154 cells/mm³	0 cells/mm³	<5 cells/mm³
Monocytes	53 cells/mm³	0 cells/mm³	<5 cells/mm³
Lactate dehydrogenase	146 U/L	78 U/L	<41 U/L
Adenosine deaminase	-	0.5 U/L	-
Gram stain	Negative	Negative	-
KOH stain	Negative	Negative	-
Ziehl–Neelsen stain	Negative	Negative	-
Bacterial culture	Negative	Negative	-
Fungal culture	Negative	Negative	-
Mycobacterial culture	Negative	Negative	-
Multiplex PCR panel for microorganisms	Negative	Negative	-
PCR for *Mycobacterium tuberculosis*	-	Negative	-
AQP4 antibodies	-	Weakly positive	-

Other autoimmune causes were ruled out through a directed autoimmune symptom review and serological testing (Table 3). The patient was discharged on oral prednisone 25 mg daily, azathioprine 50 mg every 12 hours, physical rehabilitation, and home care follow-up.

**Table 3:** Autoimmune and immunological panel AQP4, aquaporin-4; CSF, cerebrospinal fluid; Ig, immunoglobulin

Test	Result	Reference Range
Complement C3	193 mg/dL	90–180 mg/dL
Complement C4	67.5 mg/dL	10–40 mg/dL
Rheumatoid factor	<8.6 IU/mL	<14 IU/mL
Antinuclear antibodies	1:80	<1:40 or negative
Extractable nuclear antigens	0.2 AI	<1.0 AI (negative)
IgM anticardiolipin antibodies	0.6 MPL	<12 MPL (negative)
IgG anticardiolipin antibodies	1.3 GPL	<12 GPL (negative)
IgA anticardiolipin antibodies	1 APL	<20 APL (negative)
IgM anti–β2 glycoprotein I antibodies	0.6 U/mL	<20 U/mL (negative)
IgG anti–β2 glycoprotein I antibodies	1.8 U/mL	<20 U/mL (negative)
IgA anti–β2 glycoprotein I antibodies	1.3 U/mL	<20 U/mL (negative)
Lupus anticoagulant (ratio)	1.01	<1.2 (negative)
Oligoclonal bands in CSF	Negative	Negative
AQP4 antibodies in serum	Negative	Negative
Minor salivary gland biopsy	Normal histology	

At 35 weeks of gestation, the patient was admitted due to clear vaginal fluid leakage, leading to a diagnosis of premature rupture of membranes with signs of fetal distress. A cesarean section was performed, resulting in the birth of a healthy newborn with appropriate weight. After monitoring during the immediate postpartum period, the patient was discharged with instructions to continue outpatient immunosuppressive therapy and follow-up with neurology.

**Figure 2:**
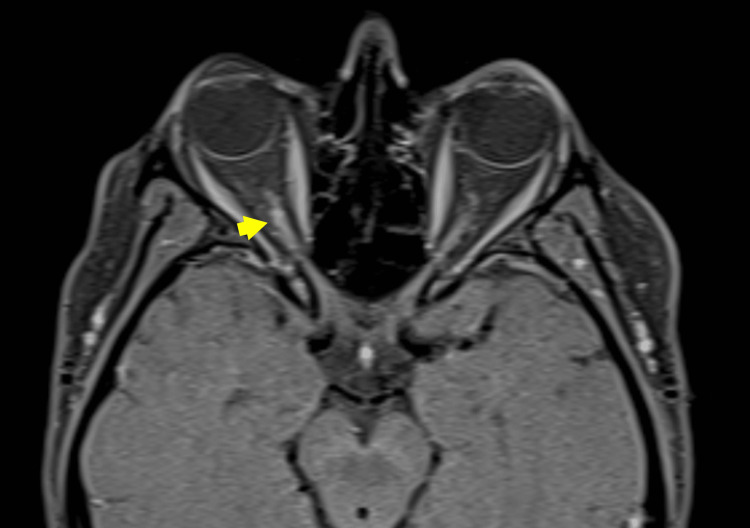
Post-contrast axial T1-weighted orbital MRI with fat suppression showing right optic neuritis. Axial post-contrast T1-weighted image with fat suppression shows abnormal enhancement and mild thickening of the right optic nerve (yellow arrowhead), consistent with acute optic neuritis.

One year and four months later, the patient presented to the emergency department with decreased visual acuity in the right eye, raising suspicion of a relapse due to right optic neuritis. She had voluntarily discontinued her medication out of concern for potential adverse effects on her breastfeeding infant. Ophthalmological examination showed visual acuity in the right eye reduced to hand motion perception, a relative afferent pupillary defect 2+, mild limitation of abduction, optic disc with blurred, elevated, and hyperemic margins, and abnormal color vision with errors on Ishihara testing and red color desaturation; biomicroscopy was unremarkable except for incipient cataract, with an intraocular pressure of 13 mmHg. In the left eye, visual acuity was 20/25 with correction, pupillary reflexes were preserved, biomicroscopy was normal, and fundus examination revealed temporal pallor of the optic nerve. Orbital MRI showed signal hyperintensity and thinning of the right intracanalicular optic nerve (Figure 2). The patient received intravenous methylprednisolone pulses without improvement in vision, prompting initiation of plasmapheresis. Three out of five planned sessions were completed, with early termination due to catheter site infection. Despite this, there was functional improvement, with visual acuity increasing from hand motion at 1 m to counting fingers at 150 cm, a reduction of the relative afferent pupillary defect from 2+ to 1+, and resolution of the marked disc edema with only slight nasal margin blurring remaining. She was discharged on azathioprine 100 mg once daily and prednisone 40 mg once daily for two weeks, with a gradual taper.

Three months later, she returned to the emergency department with recurrent decreased visual acuity in the right eye. Examination showed visual acuity reduced to hand motion, a relative afferent pupillary defect, and full extraocular movements. The anterior segment and intraocular pressure were unremarkable. Fundus examination revealed an optic disc with well-defined margins and temporal pallor of the neuroretinal rim, consistent with optic atrophy; the macula, vessels, and peripheral retina were normal. The left eye remained unchanged from the previous evaluation. This episode was considered a new relapse, attributed to administrative barriers that had prevented access to azathioprine. She was treated with intravenous methylprednisolone and discharged with instructions to resume immunosuppressive therapy and continue follow-up with neuro-ophthalmology, including computerized visual field testing.

Despite adherence to treatment, she presented four months later with lower back pain, paraparesis with gait instability, and paresthesias in both lower limbs, without ocular symptoms. This was interpreted as a new relapse due to therapeutic failure with azathioprine. She was treated with intravenous methylprednisolone followed by plasmapheresis, with good clinical response. Given the inadequate disease control, rituximab was recommended at discharge; however, the drug was not provided. Five months later, she presented with a new relapse, showing visual acuity of 20/40 in the right eye and 20/60 in the left, with marked color vision impairment (Ishihara 2/14 OD, 8/14 OS) and a mild right relative afferent pupillary defect. Anterior segment and intraocular pressure were unremarkable except for early lens opacity in the right eye. Fundus examination revealed segmental pallor of the neuroretinal rim in both eyes. Neurological examination showed left lower limb monoparesis and dorsal pain. She required a new course of plasmapheresis and was discharged on mycophenolate mofetil 500 mg every 12 hours and prednisone 50 mg daily.

**Figure 3:**
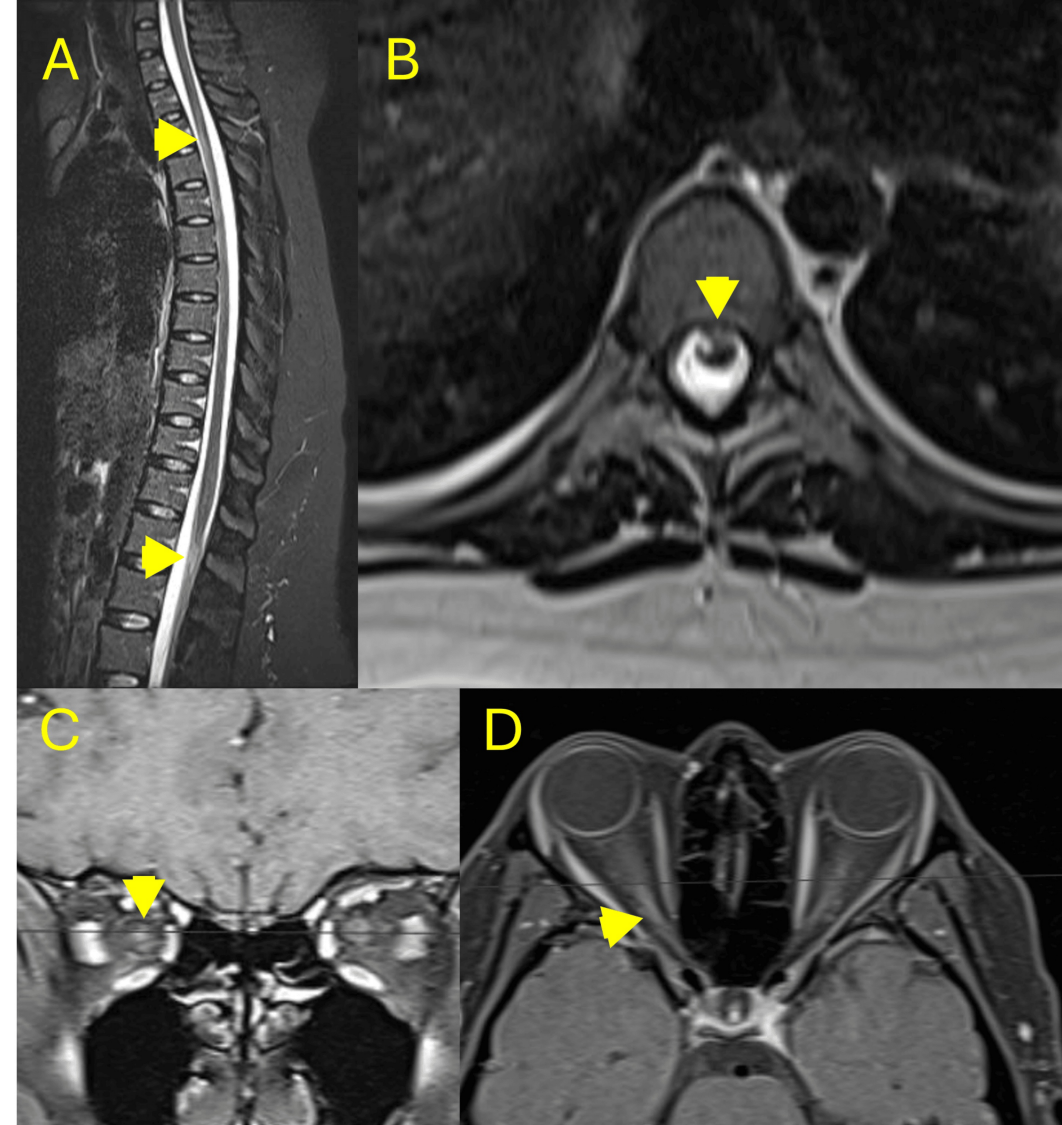
Spine and orbital MRI findings during a relapse of neuromyelitis optica spectrum disorder. A. Sagittal T2-weighted image of the thoracic spine shows a longitudinally extensive intramedullary hyperintense lesion spanning multiple vertebral segments (yellow arrowheads), consistent with transverse myelitis. B. Axial T2-weighted image at the level of the thoracic lesion reveals central spinal cord hyperintensity (yellow arrowhead). C. Coronal post-contrast T1-weighted image with fat suppression demonstrates enhancement of the optic chiasm and prechiasmatic optic nerves (yellow arrowhead). D. Axial post-contrast T1-weighted image with fat suppression shows enhancement of the left optic nerve (yellow arrowhead), consistent with unilateral optic neuritis.

The patient remained clinically stable for eight months until she presented with right lower limb paresis. Cervical, thoracic, and lumbar spine MRI revealed new hyperintense lesions in the cervicothoracic and lumbosacral segments of the spinal cord. Chronic changes were also observed in the optic nerves (Figure 3).

**Figure 4:**
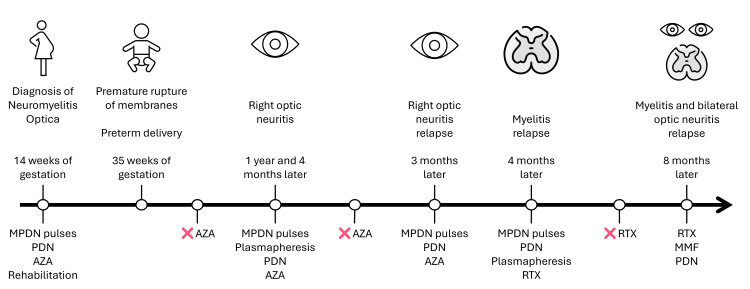
Timeline of diagnosis, obstetric events, neuro-ophthalmic relapses, immunosuppressive therapy, and treatment discontinuation in NMOSD. The figure shows the chronological visualization of obstetric events, clinical relapses, and immunosuppressive treatments following the initial diagnosis of NMOSD during pregnancy. Icons represent spinal cord and optic nerve involvement across relapses. Red Xs indicate voluntary or access-related discontinuation of therapy, often preceding clinical relapse. AZA, azathioprine; MMF, mycophenolate mofetil; MPDN, methylprednisolone; NMOSD, neuromyelitis optica spectrum disorder; PDN, oral prednisone; RTX, rituximab

Rituximab was initiated at 1,000 mg IV given in two doses two weeks apart, with maintenance infusions every six months, and mycophenolate mofetil dose was increased to 1,500 mg daily, resulting in a favorable clinical response with final visual acuity of 20/30 in the right eye and 20/60 in the left eye. Since the initiation of this regimen, the patient has not experienced further relapses, and counseling was provided to emphasize the importance of adherence and avoid treatment discontinuation. The overall clinical course, including relapses and immunosuppressive therapies, is summarized in Figure 4.

## Discussion

NMO and NMOSD are autoimmune conditions of the central nervous system characterized by recurrent episodes of optic neuritis and LETM, often associated with anti-AQP4 antibodies [1,2]. Unlike other demyelinating diseases, such as multiple sclerosis, NMOSD does not show reduced activity during pregnancy. In fact, the risk of relapse increases, particularly during the early postpartum period [1,3]. This pattern is associated with greater neurological disability and an increased risk of obstetric complications such as preeclampsia or fetal loss [3,4]. Poor outcomes during this stage are often linked to the discontinuation of immunosuppressive therapy, pregnancy-induced immunological changes, and insufficient specialized follow-up [1,5].

Current management focuses on the use of immunotherapy considered safe during pregnancy, such as azathioprine or rituximab, and aggressive treatment of relapses with corticosteroids or plasmapheresis. Outside of pregnancy, maintenance therapy with agents such as rituximab, satralizumab, or eculizumab is recommended [2,4]. A multidisciplinary approach is essential to reduce relapse frequency and preserve neurological function.

In the presented case, NMOSD led to preterm labor as an obstetric complication. Subsequently, multiple relapses occurred, ultimately resulting in optic nerve atrophy. These relapses can be attributed to disease onset during pregnancy, as well as the discontinuation and therapeutic failure of immunosuppressive therapy. Prompt recognition and treatment of each NMOSD exacerbation, along with adjustments to chronic immunosuppressive therapy, contributed to disease control and a reduction in further complications.

## Conclusions

NMOSD is a serious and potentially disabling condition that requires a high index of clinical suspicion for timely diagnosis and management, especially in young women presenting with neurological symptoms during pregnancy. This case illustrates the complexities involved in managing gestational-onset NMOSD and highlights the need for a thorough diagnostic workup, including timely neuroimaging, cerebrospinal fluid analysis, and anti-AQP4 antibody testing.

Prompt initiation of immunosuppressive therapy, such as corticosteroids, azathioprine, rituximab, or other safe alternatives, can be administered during pregnancy and lactation and is crucial for preventing relapses and long-term disability. This report also demonstrates the clinical consequences of interrupted therapy, whether due to administrative barriers or personal decisions, and reinforces the importance of multidisciplinary care, treatment continuity, and timely access to essential medications.
